# Head and Neck Cancer in Pan-American Notable People: An International Survey

**DOI:** 10.3390/dj12100305

**Published:** 2024-09-26

**Authors:** Josefina Martínez-Ramírez, Cristina Saldivia-Siracusa, Maria Eduarda Pérez-de-Oliveira, Ana Gabriela Costa Normando, Luiz Paulo Kowalski, Maria Paula Curado, Lady Paola Aristizabal Arboleda, Ana Carolina Prado-Ribeiro, Leonor-Victoria González-Pérez, Gisele Aparecida Fernandes, Florence Juana Maria Cuadra-Zelaya, Pablo Agustin Vargas, Marcio Ajudarte Lopes, Marco A. O. Magalhaes, Vidya Sankar, Alessandro Villa, Alan Roger Santos-Silva

**Affiliations:** 1 Oral Diagnosis Department, Piracicaba Dental School, University of Campinas, São Paulo 13414-903, Brazil; josefina.martinez@ues.edu.sv (J.M.-R.); c234721@dac.unicamp.br (C.S.-S.); meduardaperezo@gmail.com (M.E.P.-d.-O.); anagabicn@hotmail.com (A.G.C.N.); ana.prado@hc.fm.usp.br (A.C.P.-R.); levigope@gmail.com (L.-V.G.-P.); pavargas@fop.unicamp.br (P.A.V.); malopes@fop.unicamp.br (M.A.L.); 2 School of Dentistry, University of El Salvador, San Salvador 01101, El Salvador; florence.cuadra@ues.edu.sv; 3 Department of Head and Neck Surgery and Otorhinolaryngology, A.C. Camargo Cancer Center, São Paulo 01508-020, Brazil; lp_kowalski@uol.com.br; 4 Head and Neck Surgery Department, University of São Paulo Medical School, São Paulo 01246-903, Brazil; 5 Group of Epidemiology and Statistics on Cancer, International Research Center, A.C. Camargo Cancer Center, São Paulo 01508-020, Brazil; mp.curado@accamargo.org.br (M.P.C.); gisele.fernandes@accamargo.org.br (G.A.F.); 6 Graduate Program, A.C. Camargo Cancer Center, São Paulo 01508-020, Brazil; paola9228a@gmail.com; 7 Instituto do Câncer do Estado de São Paulo, Faculdade de Medicina, Universidade de São Paulo, São Paulo 01246-000, Brazil; 8 Oral Medicine Service, Sírio Libanês Hospital, São Paulo 01308-050, Brazil; 9 Laboratory of Immunodetection and Bioanalysis, Investigation Group POPCAD, Faculty of Dentistry, University of Antioquia, Medellín 050010, Colombia; 10 Oral Pathology and Oral Medicine, Faculty of Dentistry, University of Toronto, 124 Edward Street, Toronto, ON M5G 1G, Canada; marco.magalhaes@utoronto.ca; 11 Laboratory Medicine and Pathobiology, Faculty of Medicine, University of Toronto, 124 Edward Street, Toronto, ON M5G 1G6, Canada; 12 Sunnybrook Health Sciences Centre, Toronto, ON M4N 3M5, Canada; 13 Department of Diagnostic Sciences, Tufts School of Dental Medicine, Boston, MA 02111, USA; vidya.sankar@tufts.edu; 14 Oral Medicine, Oral Oncology and Dentistry, Miami Cancer Institute, Baptist Health South Florida, Miami, FL 33176, USA; alessandro.villa@baptisthealth.net; 15 The Herbert Wertheim College of Medicine, Florida International University, Miami, FL 33176, USA; 16 Department of Orofacial Sciences, University of California San Francisco, San Francisco, CA 94143, USA

**Keywords:** head and neck cancer, oral cancer, prevention, famous people, celebrities, public awareness

## Abstract

**Background:** The study of notable people as advocates for raising cancer awareness began in the latter decades of the 20th century. This research aimed to identify Pan-American notable people with head and neck cancer (HNC) and to explore senior health professionals’ perspectives on communicating stories of notable patients with HNC to promote prevention. **Method:** A cross-sectional survey was conducted using an online questionnaire designed in REDCap and administered to 32 senior health professionals with long-standing academic and clinical backgrounds in HNC. In addition, a structured literature review was performed on PubMed, Scopus, EMBASE, Web of Science, LILACS, and gray literature. **Results:** 18 notable figures were successfully identified from the survey, and 24 from the literature review. These individuals came from the United States, Brazil, Argentina, Mexico, El Salvador, Chile, Colombia, and Peru, and were recognized primarily for their performances as actors, artists, musicians, and athletes. The professionals’ outlooks were positive, with 31 (96.9%) agreeing that disseminating these stories can contribute to reducing risk behaviors. Furthermore, all participants (100%) agreed that such stories can promote early detection of HNC, primarily through social media, followed by the internet, and television. **Conclusions:** The study identified notable individuals and gathered positive perspectives from professionals. Our results suggest that notable people could serve as potential advocates for HNC prevention. Further research is warranted to explore the potential of this prevention strategy.

## 1. Introduction

Head and neck cancer (HNC) encompasses a heterogeneous group of tumors and continues to remain a significant public health burden worldwide, imposing substantial costs on the health system [1,2]. According to GLOBOCAN, in 2022, the number of new cancers of the oral cavity, oropharynx, and larynx in the Pan-American region was 106,080, and there were 37,256 deaths; projections indicate a progressive increase [3]. 

Apart from skin and thyroid tumors, approximately 90% of HNC cases are squamous cell carcinomas [4]. Risk factors include exposure to carcinogens from tobacco and excessive alcohol consumption [4]. Increasingly, oropharyngeal tumors are associated with previous infections with high-risk human papillomavirus (HPV), especially HPV-16 [1,4]. HNC can present with a variety of signs and symptoms. Common indicators include persistent sore throat, difficulty swallowing, lumps in the neck, red or white patches, or oral ulcerations that do not heal. Additionally, patients might experience unexplained weight loss, alterations in their voice, or a persistent cough [1,4,5]. While oral cavity cancers are easily accessible to visual inspection and could be preceded by oral potentially malignant disorders [6,7], early detection is hindered partly by the population’s limited awareness about risk factors and associated signs and symptoms extending the help-seeking behavior interval [5,8]. Despite proactive efforts by health professionals to raise awareness through various avenues, such as information materials, verbal guidance, videos, and community media campaigns, all of which have demonstrated effectiveness in increasing knowledge [9], the majority of diagnoses still are made at an advanced stage [10], which increases the risk of locoregional recurrence, distant metastasis, treatment failure, high mortality rates, and morbidity, principally in low and middle development countries [4,10].

Public interest in health issues tends to increase if a celebrity is affected by a disease [11,12,13,14,15]. Interest in the study of celebrities began in the last decades of the 20th century and continued to develop in the 21st century [16]. Celebrities (also known as notable people) are usually individuals with some degree of notability and influence in their field of activity [17]. They usually emerge from the entertainment and sports sectors, but different professions also have the power to influence a large number of people [16]. This wide recognition can be used to promote more publicity about the risk factors, signs, and symptoms of HNC [16]. While there are well-documented reports of notable people who died from complications of HNC, such as German Emperor Frederick III, US Presidents Ulysses Simpson Grant and Stephen Grover Cleveland, Austrian father of psychoanalysis Sigmund Freud, Italian composer Giacomo Puccini, baseball star George Herman “Babe” Ruth, and artist Sammy Davis [11,18,19], there is no documentation that any of them were involved in HNC prevention strategies, possibly due to limited knowledge of risk factors at the beginning of the 19th century [11]. In 2011, the announcement of Brazilian President Lula da Silva’s laryngeal cancer diagnosis led to an increase in smoking cessation consultations within a month of media coverage, demonstrating that the HNC of notable people can promote primary prevention [20]. The latter highlights the powerful impact of notable people with lived experiences of HNC who are potential advocates for prevention. Nonetheless, the current scientific landscape is characterized by a lack of studies exploring the health professionals’ perspectives on this topic.

Based on the above considerations, this study aimed to identify notable Pan-American people with HNC and explore senior health professionals’ perspectives on communicating stories of notable patients with HNC to promote prevention.

## 2. Materials and Methods

We conducted a cross-sectional study using a self-administered survey distributed among senior health professionals across Pan-American countries. Furthermore, a structured literature review was conducted to identify relevant literature documenting notable individuals with HNC from the Pan-American region.

### 2.1. Survey

The present study was approved by the Research Ethics Committee of the Piracicaba School of Dentistry (UNICAMP) and conducted in accordance with the Declaration of Helsinki’s recommendations for research involving human patients (Protocol No. 58068822.1.0000.5418). All participants signed a digital informed consent form before completing the questionnaire.

Participants were recruited by non-probability convenience sampling [21]. Professionals from esteemed national universities and public hospitals across Pan-American countries who were recognized as leaders in Oral Medicine, Oral and Maxillofacial Pathology, Head and Neck Surgery, and Clinical Oncology, with experience in research, education, or the treatment of HNC patients were eligible for inclusion. In countries where professionals with these specialties were unavailable, eligibility was expanded to include Oral and Maxillofacial Surgeons (2, 6.25%) and dentists who do not have postgraduate training (1, 3.1%) with professional experience in the fields of interest. The list of Pan-American countries is provided in Table S1.

Potential participants were identified by contacting national and international Oral Medicine and Oral Pathology organizations (Table S2) or by contacting individually. We extended formal invitations via email to forty-three potential participants across 24 countries in the region, followed by a reminder email three weeks later to improve recruitment. Thirty-two (74.4%) professionals who confirmed their participation received the survey link: https://redcap.fop.unicamp.br/redcap/surveys/?s=PEH8DPJR8D4AMWY9. Survey participants proceeded to the questionnaire only after reading and accepting the conditions of the informed consent form, available on the same link. Completed questionnaires were returned within four weeks of receiving the link. The data collection period was from 9 January to 24 October 2023. 

The questionnaire was designed using the Research Electronic Data Capture (REDCap) platform (version 13.8.1, Vanderbilt University, Nashville, TN, USA) to be distributed electronically, ensuring broad participation across the region. To ensure standardization, we opted to use the questionnaire tool in both Spanish and English. This choice aimed to guarantee uniformity in question comprehension, considering the diverse terms that might have varied meanings based on regional distinctions. The questionnaire was validated in the different languages by undergoing translation and back-translation processes. The authors (JM-R, CS-S, LPK, MPC, GAE, AV, and ARS-S) piloted the surveys before distributing them by e-mail. The survey was structured into three sections, as follows: (1) sociodemographic characteristics of the professionals (age, gender, current country of work, profession, level of academic training, and number of patients treated weekly); (2) notable people; and (3) professional perspectives. The question format was designed using categorical options and short answers. File S1 shows the questionnaires used in the study.

Data from the questionnaire were exported and organized categorically in an electronic spreadsheet using Microsoft Excel^®^ software (Microsoft 365^®^, version 2308, Microsoft Corporation, Washington, DC, USA). A descriptive statistical analysis was applied to summarize the data recorded in all questionnaire sections, using mean values, absolute numbers, and percentages.

### 2.2. Literature Review 

Individual electronic search strategies were conducted on 19 April 2022 and updated on 4 March 2024. The search strategies were done for the following databases: PubMed, Scopus, EMBASE, Web of Science, and LILACS, without period restriction. An additional search in the gray literature including Google Scholar, ProQuest, and “Biblioteca Digital de Teses e Dissertações, Brazil” was performed to identify publications regarding Pan-American notable people with HNC. The search strategy can be found in Table S3. Subsequently, studies retrieved were imported into the Endnote Web (Endnote Web, Clarivate Analytics, Philadelphia, PA, USA) for reference management, where duplicate references were systematically removed. Additionally, the reference lists of all included studies were manually screened to identify further relevant studies.

Publications were included if they met all the following criteria: (1) Publication about Pan-American notable people of any age diagnosed with HNC, defining “notable people” as researchers, physicians, scientists, inventors, journalists, actors, writers, musicians, filmmakers, politicians, activists, revolutionaries, businessmen, and athletes. (2) English, Portuguese, or Spanish language. Exclusion criteria were as follows: (1) notable people from countries other than the Pan-American region, (2) cancer in anatomical topographies different than head and neck, (3) full text is not indexed, (4) unavailable full text, (5) publication with non-specific localization of cancer, and (6) publications that did not report any notable person. 

Following the initial search, two independent reviewers performed the selection in two phases (JMR and MEPO). In the first phase, publications were selected by screening titles and abstracts using online software (Rayyan, Qatar Computing Research Institute, Available online: https://www.rayyan.ai/, accessed on 19 April 2022). Publications that met the inclusion criteria were read in full text to assess eligibility. The two reviewers continued by carefully reading the full text of the screened publication to identify the eligible publications and all the primary reasons for exclusion were registered. The study selection was always based on full-text assessment. A common consensus among the authors resolved divergences at any phase. 

Clinical and sociodemographic information for the identified notable HNC characters such as name, gender, occupation, country, anatomical site of cancer, year and age at diagnosis, risk factors, signs, and symptoms before the diagnosis, and participation as spokesperson were collected. The list of notable people was grouped by country. Data collected was tabulated and processed in Microsoft Excel^®^, and further narrative analysis was performed by descriptive statistics using absolute numbers, as well as percentages. 

## 3. Results

### 3.1. Survey 

#### 3.1.1. Sociodemographic Characteristics of Professionals 

A total of 32 senior professionals from referral centers in 22 Pan-American countries participated in the survey. Table S1 shows the distribution of participating countries. Most of the participants had postgraduate degrees in Oral Pathology (23, 71.9%) and Oral Medicine (17, 53.1%) and treated 1 to 5 HNC patients a week (20, 62.5%). Sociodemographic characteristics are detailed in Table S4. 

#### 3.1.2. Notable People Recognized by Professionals

Among the participants, 13 (40.6%) reported knowledge of 1 to 4 notable Pan-American patients with lived experiences of HNC. Their awareness of these cases was mainly through the internet (8, 32.0%), television (8, 32.0%), their own clinical practice (4, 16.0%), and information from friends, colleagues, or relatives (4, 16.0%) (Table S5).

A total of 18 notable people from the United States (5, 27.8%), Brazil (4, 22.2%), Argentina (3, 16.7%), Mexico (2, 11.1%), El Salvador (1, 5.5%), Chile (1, 5.5%), Peru (1, 5.5%), and Colombia (1, 5.5%) were identified from the survey. Of these, 15 (83.3%) were male and 3 (16.7%) were female. Regarding their occupation, artists (8, 44.4%), actors (2, 11.1%), and presidents (4, 22.2%) were the most reported. Table 1 summarizes their sociodemographic and clinical characteristics. 

#### 3.1.3. Professional Perspectives

The majority of professionals agreed that dissemination of these stories could contribute to reducing risk behaviors (31, 96.9%). Furthermore, all participants (32, 100%) agreed that they can promote early detection of HNC. The communication of stories can primarily be achieved through social media (28, 87.5%), followed by the internet (27, 84.4%) and television (25, 78.1%) (Table 2).

### 3.2. Literature Review 

#### 3.2.1. Selection Process

**Figure 1:**
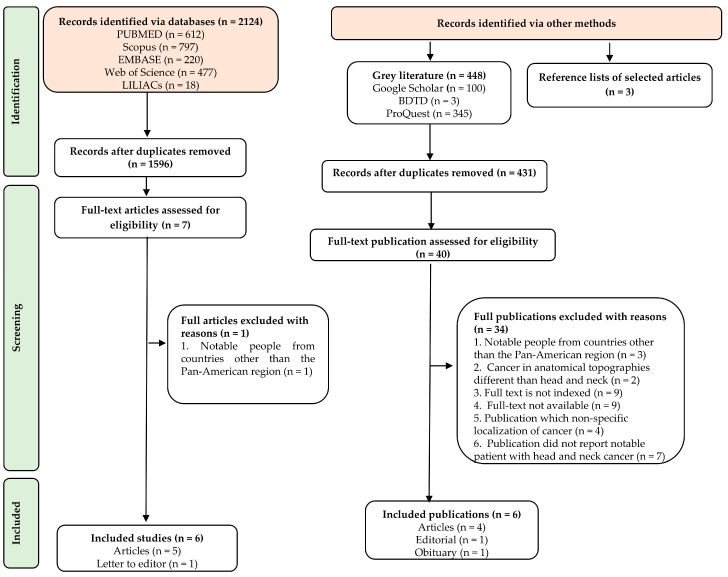
Flowchart illustrating the literature search and study selection.

The search identified 2124 records from the databases, and 448 additional from gray literature. A total of 2572 records were retrieved. In the first phase, 545 duplicates were removed, leaving 2027 to be screened by titles and abstracts. In the second phase, 47 publications that met the inclusion criteria were read as full text to assess eligibility. A total of 12 publications were selected [11,22,23,24,25,26,27,28,29,30,31,32], corresponding to 9 articles, 1 letter to the editor, 1 editorial, and 1 obituary. Additionally, three articles from the screened reference list were included. The selection publication process is presented in the flowchart (Figure 1).

#### 3.2.2. Notable People 

A total of 24 notable people from the United States (22, 91.7%), Brazil (1, 4.2%), and Argentina (1, 4.2%) were identified. Of these, 20 (83.3%) were male and 4 (16.7%) females. Regarding occupations, actors (9, 37.5%), musicians (6, 25.0%), and athletes (4, 16.7%) were the most identified. Among all, two notable people as spokespersons for cancer were reported. American singer Mary Esther Wells, diagnosed with laryngeal cancer, testified before the US Congress to encourage government funding for cancer research. American former major-league baseball player Bill Tuttle, diagnosed with oral cancer, participated as an anti-spit tobacco champion. Table 3 summarizes notable people data from the publications included. 

## 4. Discussion

This study successfully identified cases of notable individuals with HNC covered by the media or reported in the literature, shedding light on professionals’ positive perspectives regarding the potential role of these individuals in prevention efforts. To our knowledge, this is the first study conducted to identify Pan-American notable people with HNC and explore the perspective of health professionals experienced in HNC concerning the communicating stories of notable HNC patients as a preventive strategy. The data presented in this study represents an important initiative aimed at promoting the use of notable patients’ stories as a novel prevention strategy to be developed and tested in the population.

Notable people from different fields have the potential to influence a large number of laypeople on attitudes and behaviors [16,33]. A study found a positive correlation between high periods of internet searches for the most common types of cancer (e.g., breast, lung, cervix) worldwide and the advertisement of notable people diagnosed with the particular cancer reported in the media [33]. There is evidence to suggest that HNC diagnoses of notable people may promote primary prevention. For instance, in 2011, Brazilian President Lula da Silva’s laryngeal cancer, attributed to smoking, promoted policymakers to enhance the existing aggressive tobacco control agenda [20]. Moreover, there was a significant increase in smoking cessation consultations in the four weeks following the announcement of President Lula’s cancer compared to Brazil’s National No Tobacco Day or World No Tobacco Day [20]. 

A cross-database of notable people grouped by occupational domains showed that, in decreasing order, culture (30.6%), sports/games (27.7%), leadership (27.0%), and discovery/science (11.9%) were the most popular [17]. These results help explain why artists, athletes, and musicians were the professions most identified among notable people in our study. Regardless of their occupation, when a notable person is affected by a disease, the public interest in the health issue may rise [16].

Communication is essential to raising cancer awareness, disseminating cancer education (e.g., factors risk, signs, and symptoms), and reducing myths and stigma associated with cancer [34]. Large-scale public health communication campaigns through the dissemination of cancer prevention messages related to notable people’s cancer events could be an opportunity for increased awareness [35]. Prevention strategies for HNC mainly involve reducing exposure to risk factors (e.g., tobacco and alcohol consumption) and implementing large-scale HPV vaccination programs in primary prevention for HPV-related cancers [4,6]. Early detection and management are important strategies in secondary prevention, being a critical factor in the prognosis and survival of patients diagnosed with HNC. Early detection of oral cancer may be possible through oral screening due to its accessibility for visual inspection [6]. The signs and symptoms of oral and oropharyngeal cancers can be easily detected during routine clinical exams and include mouth ulcers, swelling, red or white patches, difficulty swallowing, and neck lumps [5]. In the healthcare system, dentists serve as frontline professionals in the diagnostic pathway and are often the first to notice abnormal signs or symptoms of suspicious lesions during regular oral health check-ups [36]. In addition to their diagnostic role, dentists also have the responsibility to refer patients to specialists for further evaluation and treatment. They also play a key role in educating patients about reducing the risk of HNC by promoting tobacco cessation, limiting alcohol intake, and encouraging healthy eating habits [36,37,38]. Nevertheless, patients’ limited awareness of how early and mild cancer symptoms can persist might significantly hinder them from seeking timely assistance [5]. In a previous qualitative study, males who were asked to evaluate advertisements on prostate cancer screening recommended using gender- and age-appropriate models and celebrities as spokespersons to raise awareness [39]. In our study, participants demonstrated a positive perspective on the potential of sharing reported stories of notable patients diagnosed with HNC to promote primary prevention by reducing risky behaviors and encouraging early detection. Most participants responded that stories’ communication can be channeled mainly through social media, followed by the web and television. With the widespread adoption of internet usage among the general population, it has become a significant source of health-related information [40]. Notably, news related to diseases or deaths of notable people attracts much media attention, resulting in increased media coverage, and is shared more frequently on social media [16,41]. Achieving success in social media and communications depends on sharing activities that elevate desirable health messages in people’s online social feeds [42]. Continued publicity regarding notable people’s diagnoses may encourage screening in at-risk populations and even reduce behavior risks [41,43,44]. 

This is a hypothesis-generating study that might encourage health professionals to motivate notable patients with HNC diagnoses to openly share and discuss their disease based on reliable scientific information to guarantee understanding and avoid spreading misinformation. In addition, findings presented in this paper serve as drivers of hypotheses that warrant more detailed investigation in future research studies, focusing on surveillance and measuring the impact (e.g., quality of information, circulation time, number of citations in social media, internet search, and channels of communication) of increasing public awareness following the transmission of stories of notable people with HNC.

### Strengths and Limitations

This study successfully gathers positive insights from health professionals with long-standing experiences in HNC across 22 out of 34 Pan-American countries. This broad representation is encouraging and significantly adds valuable insights to the achievement of the research’s purpose. Although responses were not obtained from all the countries, all of the most populous countries in the region were included. The non-participating countries primarily comprised small Caribbean islands. Our study has some limitations. First, the study was conducted on a small number of senior health participants from each country, thus limiting the generalization of findings to not fully represent the entire country. Second, we did not use a random selection process for target participants, as it was not considered feasible. Instead, we used a convenience sampling method to ensure representation from the Pan-American region. Future research should expand its coverage among health professionals within each country. In addition, there is a limitation in medical literature that is scarce with limited information on the sociodemographic and clinical data of the published notable patients with lived experiences in HNC. 

## 5. Conclusions

This study has successfully identified 18 notable individuals from the health professional survey response and 24 from the literature review, across 8 Pan-American countries. In addition, it provides valuable insights into the perspectives of senior health professionals from 22 Pan-American countries. Most participants expressed a positive perception regarding the potential impact on the population of disseminating stories about notable patients with HNC, leveraging communication strategies that could help to reduce risk behaviors and promote early detection. More studies are needed to further explore the potential of this strategy.

## Figures and Tables

**Table 1:** Results from survey responses regarding head and neck cancer characteristics of the Pan-American notable people.

N	Patients Name	Country	Occupation	Approximated Year of Cancer Diagnoses	Type of Cancer	Anatomical Site of Cancer ^¶^	Outcome after Diagnosis and/or Treatment
1	Michael Douglas	US	Actor	-	Carcinoma	Oropharynx HPV-mediated (p + 16)	^1^
2	Grant Achatz	US	Chef	-	Carcinoma	Lip and oral cavity	Does not know
3	Stanley Tucci	US	Artist	-	Carcinoma	Lip and oral cavity	^1^
4	Val Kilmer	US	Actor	-	Carcinoma	Oropharynx HPV-mediated (p + 16)	Does not know
5	Ebert	US	Writer/Author	-	Carcinoma	Does not know	Death
6	Luiz Inácio Lula da Silva	Brazil	President	2010	Carcinoma	Larynx	^1^
7	Heloisa Pericé	Brazil	Artist	2018	Adenocarcinoma	Other ^§^	^1^
8	Branco Melo	Brazil	Artist	2019	Carcinoma	Hypopharynx	^1^
9	Guilherme Lemos	Brazil	Researcher, artist	2015	Carcinoma	Oropharynx HPV-mediated (p + 16)	^1^
10	René Orlando Houseman	Argentina	Athlete	2018	Carcinoma	Lip and oral cavity	Death
11	Juan Jose Antonio Castelli	Argentina	President	1810	Carcinoma	Lip and oral cavity	Death
12	Gustavo Garzón	Argentina	Artist	2012	Carcinoma	Lip and oral cavity	^1^
13	Roberto D’Aubison	El Salvador	Politician	-	Carcinoma	Larynx	Death
14	Irma Lozano	Mexico	Artist	-	Carcinoma	Major salivary glands	Death
15	Lázaro Cárdenas	Mexico	President	1970	Melanoma	Other ^‡^	^2^
16	Pablo Krögh	Chile	Artist	2012	Carcinoma	Lip and oral cavity	Death
17	Alberto Fujimori	Peru	President	1997	Carcinoma	Lip and oral cavity	^2^
18	Martha Liliana Ruiz	Colombia	Artist	2021	Carcinoma	Lip and oral cavity	^1^

^¶^ According to 8th Edition AJCC Cancer Staging Manual. ^§^ Minor salivary gland oral cavity. ^‡^ Skin. ^1^ After treatment, the disease was controlled. ^2^ After treatment, the disease recurred (disease persisted).

**Table 2:** Assessment of professionals’ perspectives (*n* = 32).

Questions	*n* (%)
Communicating reported stories of notable patients diagnosed with head and neck cancer can have a positive impact on the population and promote primary prevention by reducing risky behaviors	
Yes	31 (96.9%)
No	0 (0.0%)
Does not know	1 (3.1%)
Does not answer	0 (0.0%)
Communicating reported stories of notable patients diagnosed with head and neck cancer can have a positive impact on the population and promote early detection by encouraging seeking professional care for evaluation	
Yes	32 (100%)
No	0 (0.0%)
Does not know	0 (0.0%)
Does not answer	0 (0.0%)
The population’s generated impact by news of cancer diagnoses of notable patients is short-lived	
Yes	11 (34.4%)
No	10 (31.3%)
Does not know	11 (34.4%)
Does not answer	0 (0.0%)
Dissemination of information about head and neck cancer in relation to the diagnosis of notable patients can be done by:	
Social media	28 (87.5%)
Internet	27 (84.4%)
Television	25 (78.1%)
Educational videos in healthcare centers	21 (65.6%)
Educational lectures in healthcare centers	20 (62.5%)
Newspapers	20 (62.5%)
Educational programs in dentistry schools	20 (65.5%)
Dentists	17 (53.1%)
Radio	16 (50.0%)
Educational campaigns	15 (46.9%)
Educational bulletins	11 (34.4%)
Scientific literature	11 (34.4%)
Health campaigns	7 (21.9%)
Other	0 (0.0%)
Does not know	0 (0.0%)
Does not answer	0 (0.0%)

**Table 3:** Pan-American notable people diagnosed with head and neck cancer collected from the literature review.

N	Name	Gender	Occupation	Country	Anatomical Site of Cancer ^¶^	Year of Cancer Diagnostic	Age	Signs and Symptoms before Diagnostic	Risk Factor	Spokesperson
1	Ulysses Simpson Grant	M	President	US	Oral	1884	62	Odynophagia Neck node	Smoking and alcohol consumption	-
2	Grover Cleveland	M	President	US	Oral	1893	56	Ulcer on his left hard palate	Smoking and chewing tobacco	-
3	Babe Ruth	M	Baseball player	US	Nasopharynx	1946	51	Hoarseness, and left-sided retro-orbital pain	Alcohol and tobacco consumption	-
4	Jack Klugman	M	Actor	US	Larynx	-	-	-	-	-
5	Ed Sullivan	M	Television host	US	Oropharynx	-	-	-	-	-
6	Sammy Davis Jr	M	Singer, dancer, and actor	US	Laryngeal	-	-	-	Smoking and alcohol consumption	-
7	Mary Esther Wells	F	Singer	US	Laryngeal	-	-	-	Smoking	Yes
9	Lana Turner	F	Actress and model	US	Throat	-	-	-	-	-
10	Bill Tuttle	M	Baseball player	US	Oral	-	-	Mouth sore Swelling in his cheek	Smokeless tobacco	Yes
11	George Harrison	M	The Beatles’ lead guitarist	US	Throat	-	-	-	-	-
12	Edie van Halen	M	Rock musician	US	Tongue	-	-	-	-	-
13	Michael Douglas	M	Actor	US	Oropharynx	-	-	-	-	-
14	Rusell Means	M	Actor	US	Oral	-	-	-	-	-
15	Val Kilmer	M	Actor	US	Larynx	-	-	-	-	-
16	William Hanna	M	Animator and cartoonist	US	Larynx	-	-	-	-	-
17	Khaterine Hepburn	F	Actress	US	Oropharynx	-	-	-	-	-
18	Jonh Steele	M	Paratrooper	US	Larynx	-	-	-	-	-
19	Roger Ebert	M	Film reviewer	US	Salivary gland	-	-	-	-	-
20	Dexter Keith Gordon	M	Jazz musician	US	Larynx	-	-	-	Smoking and alcohol	-
21	Amanda Blake	F	Actress	US	Throat	-	-	-	-	-
22	Lon Chaney	M	Actor	US	Throat	-	-	-	-	-
23	Lula Silva	M	President	Brazil	Larynx	2011	-		Smoking	-
24	René Houseman	M	Footballer	Argentina	Tongue	-	-	-	-	-

^¶^ According to source.

## Data Availability

Data supporting the findings of this study are available in the Supplementary Materials and from the corresponding author upon reasonable request.

## Supplementary Materials

The following supporting information can be downloaded at: https://www.mdpi.com/article/10.3390/dj12100305/s1, Table S1: Pan-American countries (n = 34) and countries participating in this study. Table S2: Name, acronym, and social networks of the Associations/Federations/Societies. Table S3: Search strategies in the databases and grey literature. Table S4: Sociodemographic characteristics of the survey’s professionals (n = 32). Table S5: Information about notable people recognized by professionals. References S1: References of included studies. File S1: Questionnaire in English and Spanish.
