# Supplementary material for: Head and Neck Cancer in Pan-American Notable People: An International Survey
Source: Dent J (Basel). 2024 Sep 26;12(10):305. doi: 10.3390/dj12100305 (PMC11505888; doi:10.3390/dj12100305)
Supplement: Supplementary file 1 [file dentistry-12-00305-s001.zip › dentistry-3180029-supplementary.pdf]

Head and Neck Cancer in Pan-American Notable People: An International Survey

Josefina Martínez-Ramírez, Cristina Saldivia-Siracusa, Maria Eduarda Pérez-de-Oliveira, Ana Gabriela Costa Normando, Luiz Paulo Kowalski, Maria Paula Curado, Lady Paola Aristizabal Arboleda, Ana Carolina Prado-Ribeiro, Leonor-Victoria González-Pérez, Gisele Aparecida Fernandes, Florence Juana Maria Cuadra-Zelaya, Pablo Agustin Vargas, Marcio Ajudarte Lopes, Marco A. O. Magalhaes, Vidya Sankar, Alessandro Villa and Alan Roger Santos-Silva

Table S1. Pan-American countries (n=34) and countries participating in this study.

#	Country	Participation
	**Caribbean**	
1	Bahamas	NP
2	Barbados	NP
3	Cuba	NP
4	Dominican Republic	P
5	France, Guadeloupe	NP
6	France, Martinique	NP
7	Haiti	NP
8	Jamaica	P
9	Puerto Rico	NP
10	Saint Lucia	NP
11	Trinidad and Tobago	NP
	**Central America**	
12	Belize	P
13	Costa Rica	P
14	El Salvador	P
15	Guatemala	P
16	Honduras	P
17	Mexico	P
18	Nicaragua	P
19	Panama	P
	**South America**	
20	Argentina	P
21	Bolivia, Plurinational State of	P
22	Brazil	P
23	Chile	P
24	Colombia	P
25	Ecuador	P
26	French Guiana	NP
27	Guyana	NP
28	Paraguay	P
29	Peru	P
30	Suriname	NP
31	Uruguay	P
32	Venezuela, Bolivarian Republic of	P
	**Northern America**	
33	Canada	P
34	United States of America	P

Source: DATA & METHODS – IARC/WHO. (2020) https://gco.iarc.fr/today/data-sources-methods

P: Participant; NP: No participation

Table S2. Name, acronym, and social networks of the Associations/Federations/Societies.

COUNTRY	NAME	ACRONYM	CONTACT / SOCIAL MEDIA
Brazil	Brazilian Society of Stomatology and Oral and Maxillofacial Pathology	SOBEP	estomatologia@sobep.com.br "@sobep"
Colombia	Colombian Academy of Oral Pathology	ACPO	patologiaoralcolombia@gmail.com patologos_orales_acpo
Chile	Chilean Society of Bucomaxillofacial Pathology	SPBMFCH	contacto@patologiaoraldhile.cl patologia.oral.de.chile
Mexico	Mexican Association of Pathology and Oral Medicine	AMPMB	ampmbcolegio@gmail.com
Nicaragua	International Oral Medicine Society	SMOI	sociedademedicinaoral@gmail.com
Paraguay	Paraguayan Society of Pathology and Oral Medicine	SPPMB	Sppmb@secretaria@gmail.com
Peru	Peruvian Association of Pathology and Oral and Maxillofacial Medicine	APPSMED	wilson.delgado@upch.pe
Uruguay	Uruguayan Society of Stomatological Pathology	SUPE	svcelhay@adinet.com.uy
Venezuela	Venezuelan Society of Oral Medicine	SVPB	@svmedicinabucal

Table S3: Search strategies in the databases and grey literature.

Database	Search strategy<br>Search data: April 19th, 2022<br>Update: March 4th 2024	Results
PUBMED	("famous persons"[MeSH Terms] OR "famous persons" OR "famous person" OR "famous people" OR notorious OR celebrity OR celebrities OR notable OR "well-known" OR "well known" OR distinguished OR remarkable OR historical OR renowned) **AND** ("Latin America"[MeSH Terms] "Latin America" OR "Latin American" OR "Pan America" OR "Pan American" OR "Hispanic or Latino"[MeSH Terms] OR Hispanic OR Hispanics OR Latino OR Latinos OR Latina OR Latinas OR American) **AND** (neoplasms[MeSH Terms] OR neoplasms OR neoplasm OR cancer OR neoplasia OR neoplasias OR malignancy OR malignancies OR malignant OR tumor OR tumors OR tumour OR tumours OR cancer OR cancers OR carcinoma OR carcinomas OR sarcoma OR sarcomas OR lymphoma OR lymphomas OR melanoma OR melanomas) **AND** (mouth[MeSH Terms] OR mouth OR oral OR lip OR lips OR palate OR "buccal mucosa" OR "floor of mouth" OR "mouth floor" OR gingiva OR oropharynx[MeSH Terms] OR oropharynx OR oropharynxs OR oropharyngeal OR "head and neck" OR thyroid OR larynx OR pharynx OR nasal OR nasopharynx OR throat OR esophagus)	612
SCOPUS	TITLE-ABS-KEY("famous persons" OR "famous person" OR "famous people" OR notorious OR celebrity OR celebrities OR notable OR "well-known" OR "well known" OR distinguished OR remarkable OR historical OR renowned) **AND** TITLE-ABS-KEY("Latin America" OR "Latin American" OR "Pan America" OR "Pan American" OR Hispanic OR Hispanics OR Latino OR Latinos OR Latina OR Latinas OR American) **AND** TITLE-ABS-KEY(neoplasms OR neoplasm OR cancer OR neoplasia OR neoplasias OR malignancy OR malignancies OR malignant OR tumor OR tumors OR tumour OR tumours OR cancer OR cancers OR carcinoma OR carcinomas OR sarcoma OR sarcomas OR lymphoma OR lymphomas OR melanoma OR melanomas) **AND** TITLE-ABS-KEY(mouth OR oral OR lip OR lips OR palate OR "buccal mucosa" OR "floor of mouth" OR "mouth floor" OR gingiva OR oropharynx OR oropharynxs OR oropharyngeal OR "head and neck" OR thyroid OR larynx OR pharynx OR nasal OR nasopharynx OR throat OR esophagus)	797

EMBASE	('famous persons'/de OR 'famous person'/de OR 'famous people'/de OR notorious OR 'celebrity'/de OR 'celebrities'/de OR notable OR 'well-known' OR 'well known' OR distinguished OR remarkable OR historical OR renowned) AND ('latin america'/de OR 'latin american'/de OR 'pan america' OR 'pan american' OR 'hispanic'/de OR 'hispanics'/de OR 'latino'/de OR latinos OR 'latina'/de OR latinas OR 'american'/de) AND ('neoplasms'/de OR 'neoplasm'/de OR 'neoplasia'/de OR neoplasias OR 'malignancy'/de OR 'malignancies'/de OR malignant OR 'tumor'/de OR 'tumors'/de OR 'tumour'/de OR tumours OR 'cancer'/de OR 'cancers'/de OR 'carcinoma'/de OR carcinomas OR 'sarcoma'/de OR sarcomas OR 'lymphoma'/de OR 'lymphomas'/de OR 'melanoma'/de OR melanomas) AND ('mouth'/de OR oral OR 'lip'/de OR 'lips'/de OR 'palate'/de OR 'buccal mucosa'/de OR 'floor of mouth' OR 'mouth floor'/de OR 'gingiva'/de OR 'oropharynx'/de OR oropharynxs OR oropharyngeal OR 'head and neck' OR 'thyroid'/de OR 'larynx'/de OR 'pharynx'/de OR nasal OR 'nasopharynx'/de OR 'throat'/de OR 'esophagus'/de)	220
Web of Science	TS=("famous persons" OR "famous person" OR "famous people" OR notorious OR celebrity OR celebrities OR notable OR "well-known" OR "well known" OR distinguished OR remarkable OR historical OR renowned) **AND** TS=("Latin America" OR "Latin American" OR "Pan America" OR "Pan American" OR Hispanic OR Hispanics OR Latino OR Latinos OR Latina OR Latinas OR American) **AND** TS=(neoplasms OR neoplasm OR cancer OR neoplasia OR neoplasias OR malignancy OR malignancies OR malignant OR tumor OR tumors OR tumour OR tumours OR cancer OR cancers OR carcinoma OR carcinomas OR sarcoma OR sarcomas OR lymphoma OR lymphomas OR melanoma OR melanomas) **AND** TS=(mouth OR oral OR lip OR lips OR palate OR "buccal mucosa" OR "floor of mouth" OR "mouth floor" OR gingiva OR oropharynx OR oropharynxs OR oropharyngeal OR "head and neck" OR thyroid OR larynx OR pharynx OR nasal OR nasopharynx OR throat OR esophagus)	477
BVS (LILACS, IBECS, BBO-ODONTOLOGIA, PAHO-IRIS)	("famous persons" OR "pessoas famosas" OR "personajes") **AND** ("head and neck neoplasms" OR "neoplasias de cabeça e pescoço" OR "neoplasias de cabeza y cuello")	18
**LITERATURA CINZENTA**		
GOOGLE SCHOLAR	("oral cancer" OR "head and neck cancer" OR "oropharyngeal cancer") **AND** ("celebrities")	100

BIBLIOTECA DIGITAL DE TESES E DISSERTAÇÃO (BDTD)	(famosos) **AND** (câncer)	3
ProQuest	TI,AB(“famous persons” OR “famous person” OR “famous people” OR notorious OR celebrity OR celebrities OR notable OR “well-known” OR “well known” OR distinguished OR remarkable OR historical OR renowned) **AND** TI,AB(“Latin America” OR “Latin American” OR “Pan America” OR “Pan American” OR Hispanic OR Hispanics OR Latino OR Latinos OR Latina OR Latinas OR American) **AND** TI,AB(neoplasms OR neoplasm OR cancer OR neoplasia OR neoplasias OR malignancy OR malignancies OR malignant OR tumor OR tumors OR tumour OR tumours OR cancer OR cancers OR carcinoma OR carcinomas OR sarcoma OR sarcomas OR lymphoma OR lymphomas OR melanoma OR melanomas) **AND** TI,AB(mouth OR oral OR lip OR lips OR palate OR “buccal mucosa” OR “floor of mouth” OR “mouth floor” OR gingiva OR oropharynx OR oropharynxs OR oropharyngeal OR “head and neck” OR larynx OR pharynx OR nasal OR nasopharynx OR throat)	345

Table S4. Sociodemographic characteristics of the survey's professionals (n=32).

Characteristics	n (%)
**Age**	
Mean	45 years
Range	29 -82 years
**Gender**	
Female	16 (50.0%)
Male	16 (50.0%)
**Country**	
Mexico	4 (12.5%)
Argentina	2 (6.25%)
Chile	2 (6.25%)
Colombia	2 (6.25%)
Dominican Republic	2 (6.25%)
El Salvador	2 (6.25%)
Peru	2 (6.25%)
United States	2 (6.25%)
Brazil	1 (3.1%)
Canada	1 (3.1%)
Costa Rica	1 (3.1%)
Ecuador	1 (3.1%)
Guatemala	1 (3.1%)
Honduras	1 (3.1%)
Paraguay	1 (3.1%)
Uruguay	1 (3.1%)
Venezuela	1 (3.1%)
Belize	1 (3.1%)
Bolivia	1 (3.1%)
Jamaica	1 (3.1%)
Nicaragua	1 (3.1%)
Panama	1 (3.1%)
**Profession**	
Dentist	30 (93.8%)
Physician	2 (6.25%)
**Specialist**	
Oral Pathology	12 (37.5%)
Oral Medicine	6 (18.8%)
Oral Maxillofacial Surgery	2 (6.25%)
Head and Neck Surgery	1 (3.1%)
Oncologist Clinic	1 (3.1%)
**Master's Degree**	
Oral Pathology	6 (18.8%)
Oral Medicine	3 (9.4%)
Other*	2 (6.25%)
**Doctorate Degree**	
Oral Pathology	5 (15.6%)
Oral Medicine	8 (25.0%)
**Does no formal postgraduate preparation**	1 (3.1%)
**Number of patients treated per week**	
Does not treat	2 (6.25%)
Less than 5 per week	20 (62.5%)
5 or 10 per week	1 (3.1%)

15 to 20 per week	1 (3.1%)
More than 20 per week	1 (3.1%)
Does not known	1 (3.1%)
Prefer not to answer	6 (18.8%)

Table S5. Information about notable people recognized by professionals.

Questions	n (%)
**Do you know any Pan-American notable patient with current or previously diagnosed head and neck cancer whose case was covered by the media?** (n=32)	
Yes	13 (40.6%)
No	19 (59.4%)
**How many?** (n=13)	
1	5 (38.5%)
2	5 (38.5%)
3	2 (13.4%)
4	1 (7.7%)
>4	0 (0.0%)
**How did you find out about the notable patient's disease?** (n=25)	
Internet	8 (32.0%)
Television	8 (32.0%)
Friends/colleagues/relatives	4 (16.0%)
Social media	1 (4.0%)
Scientific literature	0 (0.0%)
Radio	0 (0.0%)
Newspapers	0 (0.0%)
Other*	4 (16.0%)

* Other: Own patient with press coverage.

File S1. Questionnaire in English and Spanish.

Note: Responses to questions 1 and 4 were not analyzed in this study.

QUESTIONNAIRE IN ENGLISH

SECTION 1: DEMOGRAPHIC ASPECTS

1. **Date of birth:**________________
2. **Age:**_______________ years
3. **Gender:**____________
4. **Country of birth:** ___________________________
5. **Country where you currently work in:** ______________________
6. **Profession:** ( ) Physician ( ) Dentist ( ) Other
7. **Last level of training obtained:**
   ( ) Specialist
   ( ) Oral Pathology ( ) Stomatology/Oral Medicine ( ) Head and Neck Surgeon ( ) Radio-oncologist ( ) Clinical oncologist ( ) Other: ______
   ( ) Master's Degree
   ( ) Oral Pathology ( ) Stomatology/Oral Medicine ( ) Head and Neck Surgeon ( ) Radio-oncologist ( ) Clinical oncologist ( ) Other: ______
   ( ) Doctorate
   ( ) Oral Pathology ( ) Stomatology/Oral Medicine ( ) Head and Neck Surgeon ( ) Radio-oncologist ( ) Clinical oncologist ( ) Other: ______
   ( ) I did not obtain formal post-grade preparation on the field, but I have developed experience through years of professional practice.
   Years:____
8. **How often do you treat patients with head and neck cancer?**
   Number of patients:_____________ per week
   Does not know
   Prefer not to answer

SECTION 2: NOTORIOUS PATIENTS DIAGNOSED WITH HEAD AND NECK CANCER

1. **Do you know any Pan-American notorious patient with current or previously diagnosed head and neck cancer whose case was covered by the media?**
   ( ) Yes
   ( ) No

2. **How many?**
   ( ) 1
   ( ) 2
   ( ) 3
   ( ) 4
   ( ) >4

3. **Name patient: _________**

4. **Country of origin:_______**

5. **Why is he/she known for?**
   ( ) Athlete
   ( ) Artist
   ( ) Songwriter
   ( ) Writer/Author
   ( ) Physician
   ( ) Business
   ( ) Social media influencer
   ( ) Governor
   ( ) Researcher
   ( ) Politician
   ( ) Other
   ( ) Does not known
   ( ) Does not answer

6. **Do you know the approximate year of diagnosis?**
   ( ) Yes
   ( ) No

   Year: _____

7. **Primary tumor localization:**
   ( ) Lip and oral cavity
   ( ) Major salivary gland
   ( ) Nasopharynx
   ( ) Oropharynx HPV-mediated (p16+)
   ( ) Oropharynx (p16-) and hypopharynx

( ) Nasal cavity and paranasal sinuses
( ) Larynx
( ) Mucosal melanoma of the head and neck
( ) Cutaneous squamous cell carcinoma of the head and neck
( ) Other
( ) Does not known
( ) Does not answer

8. **Tumor type:**
( ) Carcinoma
( ) Sarcoma
( ) Lymphoma
( ) Adenocarcinoma
( ) Melanoma
( ) Metastases
( ) Does not known
( ) Does not answer

9. **Outcome after diagnosis and/or treatment: ____________________**

10. **How did you find out about the notorious patient's disease?**
( ) Scientific literature
( ) Radio
( ) Television
( ) Internet
( ) Social media
( ) Newspaper
( ) Friends/colleagues/relatives
( ) Other
( ) Does not known
( ) Does not answer

SECTION 3: PROFESSIONAL'S PERSPECTIVES

1. **Communicating reported stories of notorious patients diagnosed with head and neck cancer can have a positive impact on the population and promote primary prevention by reducing risk behaviors.**
( ) Yes
( ) No

( ) Does nor know
( ) Does not answer

2. **Communicating reported stories of notorious patients diagnosed with head and neck cancer can have a positive impact on the population and promote early detection by encouraging seeking professional care for evaluation.**
( ) Yes
( ) No
( ) Does not know
( ) Does not answer

3. **The population's generated impact by the news pf cancer diagnosis of notorious patients is short-lived.**
( ) Yes
( ) No
( ) Does not know
( ) Does not answer

4. **Dissemination of information about head and neck cancer in relation to the diagnosis of notorious patients can be done by:**
( ) Scientific literature
( ) Television
( ) Internet
( ) Radio
( ) Social media
( ) Newspaper
( ) Educational videos in healthcare centers
( ) Educational programs in dental schools
( ) Educational bulletins
( ) Health campaigns
( ) Dentists
( ) Other
( ) Does not know
( ) Does not answer

CUESTIONARIO EN IDIOMA ESPAÑOL

SECCIÓN 1: ASPECTOS DEMOGRÁFICOS

1. **Fecha de nacimiento:________________**
2. **Edad:____________**en años.
3. **Género:**
4. **País de nacimiento:**
5. **País donde ejerce su profesión actualmente:**
6. **Profesión:** ( ) Médico ( ) Odontólogo ( ) Otro
7. **Último nivel de formación obtenido:**
   ( ) Especialista
   ( ) Patología Oral ( ) Estomatología ( ) Cirujano de Cabeza y Cuello ( ) Radio-oncólogo
   ( ) Oncólogo Clínico ( ) Otro: ________
   ( ) Maestría
   ( ) Patología Oral ( ) Estomatología ( ) Cirujano de Cabeza y Cuello ( ) Radio-oncólogo
   ( ) Oncólogo Clínico ( ) Otro: ________
   ( ) Doctorado
   ( ) Patología Oral ( ) Estomatología ( ) Cirujano de Cabeza y Cuello ( ) Radio-oncólogo
   ( ) Oncólogo Clínico ( ) Otro: ________
   ( ) No tengo una preparación formal de posgrado en el área, pero he desarrollado experiencia durante años de práctica profesional.
   Años: ________
8. **¿Con qué frecuencia usted trata pacientes con cáncer de cabeza y cuello?**
   Numero de paciente: ________ por semana
   ( ) No sabe
   ( ) Prefiere no responder

SECCIÓN 2: PACIENTES NOTORIOS DIAGNOSTICADOS CON CÁNCER DE CABEZA Y CUELLO

1. **¿Conoce algún personaje notorio Panamericano con diagnóstico actual o anterior de cáncer de cabeza y cuello cuyo caso fue divulgado en medios de comunicación?**
   ( ) Sí
   ( ) No

2. **Cuantos?**

( ) 1
( ) 2
( ) 3
( ) 4
( ) >4

3. **Nombre Personaje:_________**

4. **País de origen: ___________**

5. **¿Por qué es conocido?**
( ) Atleta
( ) Artista
( ) Compositor
( ) Escritor
( ) Empresario
( ) Influencer en redes sociales
( ) Médico
( ) Gobernante
( ) Investigador
( ) Político
( ) Otro
( ) No sabe
( ) No responde

6. **¿Conoce aproximadamente en qué año fue diagnosticado?**
( ) Sí
( ) No

Año: ______

7. **Tipo de tumor:**
( ) Carcinoma
( ) Sarcoma
( ) Linfoma
( ) Adenocarcinoma
( ) Melanoma
( ) Metástasis

( ) No sabe
( ) No responde

8. Localización de tumor primario:
( ) Labio y cavidad bucal
( ) Glándulas salivares mayores
( ) Nasofaringe
( ) Orofaringe VPH-positivo (p16+)
( ) Orofaringe VPH-negativo (p16-) e hipofaringe
( ) Cavidad nasal y senos paranasales
( ) Laringe
( ) Melanoma de la mucosa de cabeza y cuello
( ) Carcinoma espinocelular cutáneo de cabeza y cuello
( ) Otro
( ) No sabe
( ) No responde

9. Evolución posterior al diagnóstico y/o tratamiento:
( ) Después del tratamiento, la enfermedad se controló
( ) Después del tratamiento, presento recidiva (la enfermedad persistió)
( ) Muerte
( ) No sabe
( ) No responde

10. ¿Como se enteró de la enfermedad del paciente notorio?
( ) Literatura científica
( ) Radio
( ) Televisión
( ) Internet
( ) Redes sociales
( ) Revistas
( ) Amigos/colegas/parientes
( ) Otros
( ) No sabe
( ) No responde

SECCIÓN 3: PERSPECTIVAS DE LOS PROFESIONALES

1. **La comunicación de historias reportadas de paciente notorios diagnosticados con cáncer de cabeza y cuello puede tener un impacto positivo en la población y promover la prevención primaria reduciendo los comportamientos de riesgo.**
( ) Sí
( ) No
( ) No sabe
( ) No responde

2. **La comunicación de historias reportadas de pacientes con cáncer de cabeza y cuello puede tener un impacto positivo en la población y promover la detección temprana por medio de la búsqueda de atención profesional para evaluación.**
( ) Sí
( ) No
( ) No sabe
( ) No responde

3. **El impacto que genera en la población la noticia del diagnóstico de cáncer de un paciente notorio es de corta duración.**
( ) Sí
( ) No
( ) No sabe
( ) No responde

4. **La difusión de la información sobre el cáncer de cabeza y cuello en relación con los diagnósticos de los pacientes notorios puede ser transmitida a la populación a través de:**
( ) Literatura científica
( ) Televisión
( ) Internet
( ) Radio
( ) Redes sociales
( ) Revistas
( ) Vídeo educativos en centros de atención de salud
( ) Conferencias educativas en centros de atención de salud
( ) Programas educativos en las facultades de odontología
( ) Boletines educativos
( ) Campañas educativas
( ) Campañas de salud

( ) Dentistas
( ) Otro
( ) No sabe
( ) No responde
